# Impact of volume and expression time in an AAV-delivered channelrhodopsin

**DOI:** 10.1186/s13041-023-01067-1

**Published:** 2023-11-10

**Authors:** Sanaz Ansarifar, Gabija Andreikė, Milad Nazari, Rodrigo Labouriau, Sadegh Nabavi, Andrea Moreno

**Affiliations:** 1 Danish Institute of Translational Neuroscience (DANDRITE), Aarhus University, Aarhus, Denmark; 2 Centre for Proteins in Memory (PROMEMO), Danish National Research Foundation, Aarhus University, Aarhus, Denmark; 3 Department of Molecular Biology and Genetics, Aarhus University, Aarhus, Denmark; 4 Applied Statistics Laboratory, Department of Mathematics, Aarhus University, Aarhus, Denmark

**Keywords:** Optogenetics, In vivo electrophysiology, EPSP, oChIEF, Volume, Expression time, Longitudinal, Channelrhodopsin, AAV

## Abstract

Optogenetics has revolutionised neuroscience research, but at the same time has brought a plethora of new variables to consider when designing an experiment with AAV-based targeted gene delivery. Some concerns have been raised regarding the impact of AAV injection volume and expression time in relation to longitudinal experimental designs. In this study, we investigated the efficiency of optically evoked post-synaptic responses in connection to two variables: the volume of the injected virus and the expression time of the virus. For this purpose, we expressed the blue-shifted ChR2, oChIEF, employing a widely used AAV vector delivery strategy. We found that the volume of the injected virus has a minimal impact on the efficiency of optically-evoked postsynaptic population responses. The expression time, on the other hand, has a pronounced effect, with a gradual reduction in the population responses beyond 4 weeks of expression. We strongly advise to monitor time-dependent expression profiles when planning or conducting long-term experiments that depend on successful and stable channelrhodopsin expression.

**Supplementary Information:**

The online version contains supplementary material available at 10.1186/s13041-023-01067-1.

## Introduction

Optogenetics has brought a transformative change to neuroscience and has undoubtedly opened a wide array of possibilities in experimental design. It is now widely used by an increasing number of laboratories to manipulate circuits with high precision [1–7], particularly in combination with AAV-mediated expression systems, which expands its range of applications [4]. However, technical details of the specific protocols of AAV handling, intra-cerebral injections and expression times vary greatly across publications and are often insufficiently explained in methodological descriptions [8]. In addition to already-described serotype effects [9, 10], region-dependent expression tropisms [10], and AAV-mediated dendritic loss [11], the variability of experimental conditions adds to the uncertainty about the reproducibility of results, and especially poses a problem to researchers building an experiment from the ground up.

The most widely used blue-shifted excitatory channelrhodopsin, ChR2, has been extensively modified to create a range of variants tailored to diverse experimental needs. For instance, modifying specific functional aspects, such as accelerating its kinetics, can determine the suitability of a channelrhodopsin for delivering high-frequency stimulation patterns. Among those ChR2 variants, oChIEF is often used for high-frequency optical stimulation (for example, for the induction of long-term potentiation) due to its fast kinetics [12–14].

The lateral thalamus-amygdala pathway is widely investigated for its role in associative learning in general and in cued aversive conditioning in particular (e.g., [12, 15]). Additionally, it is often used to study the longitudinal effects of different pharmacological, physiological or behavioural manipulations (e.g., effects observed on memory permanence), which makes it an ideal candidate for studying the reliability of this optogenetic tool in evoking postsynaptic potentials under different experimental conditions.

In this study, we have evaluated the presence of fluorescence (as an indicative of viral expression) and the in vivo evoked population responses to optical stimulation under several conditions. To do this, we have injected the vector ssAAV-8/2-hSyn1-oChIEF-tdTomato(non-c.d.)-WPRE-SV40p(A) in order to express oChIEF in the lateral thalamus in a wildtype mouse model (C57BL/6J). We have compared the extent of the infection at the injected location (i.e., pre-synaptic neurons in the lateral thalamus) with the post-synaptic population responses evoked by axonal stimulation in the amygdala (i.e., post-synaptic responses elicited by incoming afferents from the lateral thalamus). This has been done at three different time points post-injection (4, 6 and 8 weeks) and four different injected volumes (0.264, 0.528, 0.793 and 1.056 μl). The details of the experimental protocol and statistical analysis can be found in Additional file 1. In short, we measured the extent of the expression area based on the presence of fluorescence; in a subset of animals, we measured the amplitude of the evoked responses to five different light intensities.

**Fig. 1:**
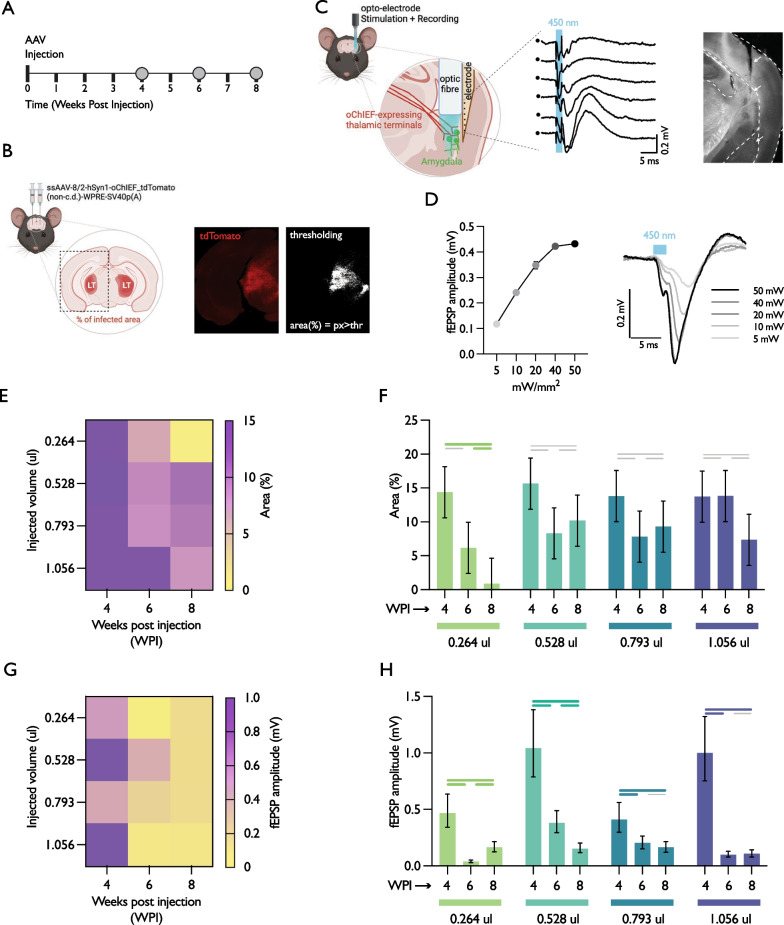
Expression time, but not injected volume, greatly impacts light-evoked potentials in the thalamus-amygdala pathway. **A** Schematic representation of the experimental timeline; **B** Example of tdTomato fluorescence tag (for AAV expression localisation) and approximate area of infection, for one hemisphere; **C** Schematic representation of the recording electrode positioning (left) and population field evoked responses based on the dorso-ventral profile observed during recording (middle) with a representative histological image showing the electrode lesion trace (right); **D** representative light-evoked fEPSP for different stimulation intensities; (E) quantification of approximate infection area based on expression time (x-axis) and injected volume (y-axis), n = 18 (n = 6 per WPI); **F** data as in **E** reorganised by expression time to show multiple comparisons across time points; **G** quantification of the amplitude of the evoked responses based on expression time and injected volume in a subset of the animals in **C**, n = 14 (4 WPI, n = 4; 6 WPI, n = 6; 8 WPI, n = 4); **H** data as in **G** reorganised by expression time to show multiple comparisons across time points. Data are represented as means and 95% coverage confidence intervals. Thick coloured lines represent statistically significant differences at a 5% level of significance. Thin grey lines represent no statistical significance detected. P-values are reported in Additional file 1: Figure S2

Figure 1 (panels 1E and 1G) show the results for expression area and evoked population responses, respectively, for all different volumes (y-axis) and weeks post injection (x-axis) analysed. In these matrices, it is possible to gauge the effect of combining the different conditions. The results of multiple comparison analyses are then shown in Fig. 1, panels 1F and 1H (for comparisons across weeks) and Additional file 1: Figure S1, panels S1A and S1B (for comparisons across injected volumes).

We found no significant differences in area across volumes at 4 or 6 WPI. At 8 WPI, the area at the lowest volume (0.264 μl) was significantly smaller than all the other volumes (0.528 μl, 0.793 μl and 1.056 μl), but no differences were found amongst the remaining volumes (Additional file 1: Figure S1A). When comparing the effect of time within each injection volume (panel 1F), we only found significant differences at 0.264 μl, where the area detected at 8 WPI is significantly smaller than the area detected at 4 and 6 WPI. All in all, we hypothesise that the differences observed at the lowest volume between 8 WPI and the other two time points could be attributed to lower amounts being more susceptible to volumetric spread differences.

In panels 1G and 1H, fEPSP measurements from a subset of the animals shown in 1E and 1F are plotted. Surprisingly, when comparing fEPSPs across time for each volume (panel 1H) we observed a significant fEPSP signal decay with time from 4 to 6 WPI in all volumes. Significant differences between 6 and 8 WPI were also observed in 0.264 μl (increase) and 0.528 μl (decrease), but not in 0.793 μl or 1.056 μl.

When comparing different injection volumes within each time point (Additional file 1: Figure S1B), we observed significant differences at 4 WPI (i.e., all volumes different from each other except 0.264 μl from 0.793 μl, and 0.528 μl from 1.056 μl) and 6 WPI (i.e., all volumes different from each other) but no differences at 8 WPI, where all signals had small amplitudes (< 0.2 mV) and were indistinguishable from each other. In general, recorded fEPSPs were qualitatively different at 4 WPI (> 0.4 mV), while at the other two later time points fEPSPs were consistently smaller (< 0.4 mV) regardless of the injected volume.

All in all, as mentioned above the observed evoked population responses were dramatically compromised after 4 weeks post-injection (see Fig. 1G and H). These findings are aligned with previous reports on the toxicity of AAVs [11] that could be building up over time and explain the lack of detected population responses. Furthermore, in experiments using freely-moving mice (data not shown) we have observed a similar temporal decay of the population responses. This points to a within-subject impact of expression time in optogenetic experiments performed in animals chronically implanted with recording devices.

Traditionally, injection volume has been one of the main variables reported in optogenetic studies, while expression time is often described with considerable variation (i.e., studies reporting a wide window between 3–8 weeks post-injection times for the start of experimental procedures). Here, we report that the duration of expression is a crucial variable that impacts the signal obtained by light-evoked electrophysiological recordings, which by extension impacts the physiological stability of the network. While this may be less relevant to experiments that are performed within a single session, it is crucial to studies that are performed longitudinally or that rely on the repeated sampling of subjects over time. The latter type of experiments necessitates guaranteeing a stable level of protein expression to ensure reliable experimental conditions and physiology. Even though this study has only been conducted by assessing the effects of a specific channelrhodopsin and synaptic pathway, these results call for caution when carrying-out longitudinal optogenetic experiments in general, and advise towards the need of performing and reporting comprehensive dose–response pilot experiments.

### Supplementary Information

**Additional file 1.** Supplementary methods and material. **Supplementary Figure S1.** Multiple comparisons between volumes on each time point shown (same data as in Figure 1 panels E and F, reorganised to show multiple comparisons). Data are represented as means and 95% coverage confidence intervals. Thick coloured lines represent statistically significant differences at a 5% level of significance. Thin grey lines represent no statistical significance detected. **Supplementary Figure S2.** *P*-values corresponding to all the comparisons shown in Figure 1 and Supplementary Figure S1. The value 0 is given to *p* < 0.001.

## Data Availability

Data are available upon request from the corresponding author.

## Abbreviations

AAV: Adeno-associated virus
DAPI: 4′,6-Diamidino-2-phenylindole
fEPSP: Field excitatory post-synaptic potential
LT: Lateral thalamus
WPI: Weeks post injection
px: Pixels
thr: Threshold
