# Supplementary material for: Impact of volume and expression time in an AAV-delivered channelrhodopsin
Source: Mol Brain. 2023 Nov 10;16:77. doi: 10.1186/s13041-023-01067-1 (PMC10638758; doi:10.1186/s13041-023-01067-1)
Supplement: Supplementary file 1 — Additional file 1. Supplementary methods and material. Supplementary Figure S1. Multiple comparisons between volumes on each time point shown (same data as in Figure 1 panels E and F, reorganised to show multiple comparisons). Data are represented as means and 95% coverage confidence intervals. Thick coloured lines represent statistically significant differences at a 5% level of significance. Thin grey lines represent no statistical significance detected. Supplementary Figure S2. P-values corresponding to all the comparisons shown in Figure 1 and Supplementary Figure S1. The value 0 is given to p < 0.001. [file 13041_2023_1067_MOESM1_ESM.docx]

# Additional file 1

# Materials and Methods

## AAV viral vector

ssAAV-8/2-hSyn1-oCHIEF_dTomato(non-c.d.)-WPRE-SV40p(A) was purchased from the Neuroscience Center Zurich (ZNZ) Viral Vector Facility (VVF) repository at a physical titer of 6.6 x 10^12^ vg/ml, diluted 1.5x and shipped in 50 ul aliquots (final concentration ≈ 4.4 x 10^12^ vg/ml). After arrival, the virus was stored at -70°C for 2 weeks before aliquoting. 10 ul aliquots were prepared and stored at -70°C until the day of injection. Aliquots were stored at 4°C for no more than 24h during injection procedures.

## Mouse preparation and stereotaxic injections

Mice (7 week old male wildtype C57BL/6J) were housed in groups of 3-4 with ad libitum access to food and water and under a 12 h light/dark cycle (lights on at 6 AM).

Mice were anaesthetised with Isoflurane (0.15-0.2 % in 0.8 l/min air) and placed in a stereotaxic frame. Hair was shaved using scissors and the skin was cleaned with Iodine solution. A local anaesthetic was applied to the incision points (0.1 ul Lidocaine, s.c.) and an incision was made to expose the cranial surface. A small trephine was drilled into the skull in the coordinates for the lateral thalamus (3.15 mm antero-posterior from bregma, 1.83 mm medio-lateral from bregma, and 3.5 mm dorso-ventral from the cranial surface), and a thin glass pipette was lowered into the goal coordinate. Viral volumes were calculated based on the internal diameter of the pipette. The virus was delivered bilaterally using a pico-injector (PLI-100A, Marsap Services) at a rate of approximately 0.1 ul/min. An additional 10 min were allowed in-place for post-injection diffusion. The skin was sutured using skin staples (Michel Suture Clips, AgnTho’s) and mice were allowed to recover on a heated pad before being brought back to the home cage. Mice were monitored daily for post-operative care and administered analgesics (buprenorphine 0.1 mg/kg, s.c.) until their complete recovery.

Mice were anaesthetised with a mixture of fentanyl (0.05 mg/kg), medetomidine (0.5 mg/kg) and midazolam (5 mg/kg) – FMM (10 ml/kg, i.p.). For histological analysis of the tissue, mice were sacrificed by transcardial perfusion (see below). In a subset of mice, electrophysiological recordings were performed before perfusion. In that case, FMM anaesthesia was followed by a local injection of anaesthetic into the incision points (0.1 ul lidocaine, s.c.) and an incision was made to expose the cranial surface. A small trephine was drilled into the skull in the coordinates for the amygdala (1.65 mm antero-posterior from bregma, 3.55 mm medio-lateral from bregma, and 3.5-3.6 mm dorso-ventral from the cranial surface), and an opto-electrode composed of a 200 um optic fibre attached to a 32-channel silicon probe (Neuronexus, Ann Arbor, Michigan) was lowered into the amygdala.

## Tissue preparation and histological imaging

Mice were sacrificed by transcardial perfusion under general anaesthesia (FMM). Brains were then harvested and stored in 4% formalin until sliced. For slicing, brains were embedded in 4% agarose and sections of 70 um were cut with a vibratome (Leica, VT1000S). Free floating slices were stained with NeuN (1:500) and Alexa 488 for marking of neuronal nuclei, and DAPI (1:1000) for anatomical localisation.

An Apotome fluorescence microscope (Axio-Imager M2), a Hamamatsu ORCA-flash 4.0 camera and an HPX 120V light source were used for all histological imaging under a 10x magnification.

## Data analysis

### Electrophysiology

﻿Raw data were filtered (0.1–3000 Hz), amplified (100x), digitised and stored (25 kHz sampling rate) for offline analysis using a Multichannel Systems data acquisition system (Multichannel Systems, Reutlingen, Germany). Offline data analysis was performed using custom routines in Spike2 and Matlab. Excitatory post-synaptic field potentials (fEPSP) were evoked by delivering short (2 ms) light pulses with a Doric LED-Diode 450 nm laser source at different intensities of stimulation (5, 10, 20, 40 and 50 mW/mm^2^, 3 pulses per intensity) with an inter-pulse-interval of 20 s. The amplitude of the light-evoked field potentials was measured as the value difference (in mV) between the baseline value (i.e., average signal of the 5 ms prior to the onset of the light stimulation) and the minimum detectable peak (3-4 ms after the light stimulation onset).

In the mice with clear fEPSPs (>0.2 mV), those were observed (and recorded) at the position of the first electrode’s penetration (i.e., at the original coordinates). Nevertheless, a certain variability in probe placement cannot be completely excluded due to putative bregma and lambda location errors, and/or biological variability. Because of this, in the instances where no clear fEPSPs were found at the original coordinates, we performed a minimum of 4 successive electrode penetrations in the 4 cardinal directions in a 200 um ratio from the original coordinate. There were no cases where signal was found with this method when the first penetration was unsuccessful, and therefore we report the data obtained at the original coordinates.

### Estimation of infection area based on fluorescence imaging

To determine the expression level accurately, we first addressed the blocking artifact resulting from Apotome during the photo capture process. We accomplished this by applying individual intensity scaling factors to each block, minimizing any noticeable changes in brightness at the block borders. To determine the appropriate scaling values, an optimization problem was formulated, considering the equality condition for the average brightness at the boundaries of neighbouring block pairs. Finally, the problem was solved using the "quadprog" function in Matlab (1). Upon successfully removing the blocking artifact, we employed a piece-wise linear mapping technique to achieve a consistent brightness distribution across different slices. This ensured that different slices had similar brightness profiles. The background brightness of each slice was set to 40, while the peak brightness was determined by identifying the brightness level surpassed by the top 0.1% of pixels within the region of interest (ROI), which was fixed at 255. To measure the expression area, a threshold equal to three times the background brightness (3 * 40 = 120) was applied to each hemisphere. The expression area was then calculated by determining the proportion of pixels whose brightness exceeded this threshold in each respective hemisphere.

### Statistical analyses

We modelled the evoked responses’ amplitudes using a generalised linear mixed model defined with the compound Poisson Gamma distribution, which allows for representing positive responses presenting zeroes (not detectable amplitudes). The models were defined using a logarithmic link function and contained a Gaussian random component representing the mice and a fixed effect representing the combination of the weeks, volumes, and light intensities.

The affected area was modelled using a generalised linear model defined with the Gamma distribution, the logarithmic link function and two explanatory classification variables representing the week and the volume. Note that only one area was determined per mouse; therefore, we used a model assuming that the observations were independent.

The adequacy of the Gamma and the Compound Poisson Gamma model described above was verified by testing the adherence to a standard uniform distribution of the responses transformed by the respective cumulative distribution functions (p-values 0.6477 and 0.3892, respectively). The additivity and the presence of effects of light, weeks, and volumes were tested using likelihood ratio tests in both models.

All the models were adjusted with the software R (2) using the R-package “lme4” (3). Post-hoc analyses were performed using the R-package “postHoc” (4), with p-values adjusted by the false discovery rate (5) for correcting for multiple testing.

#
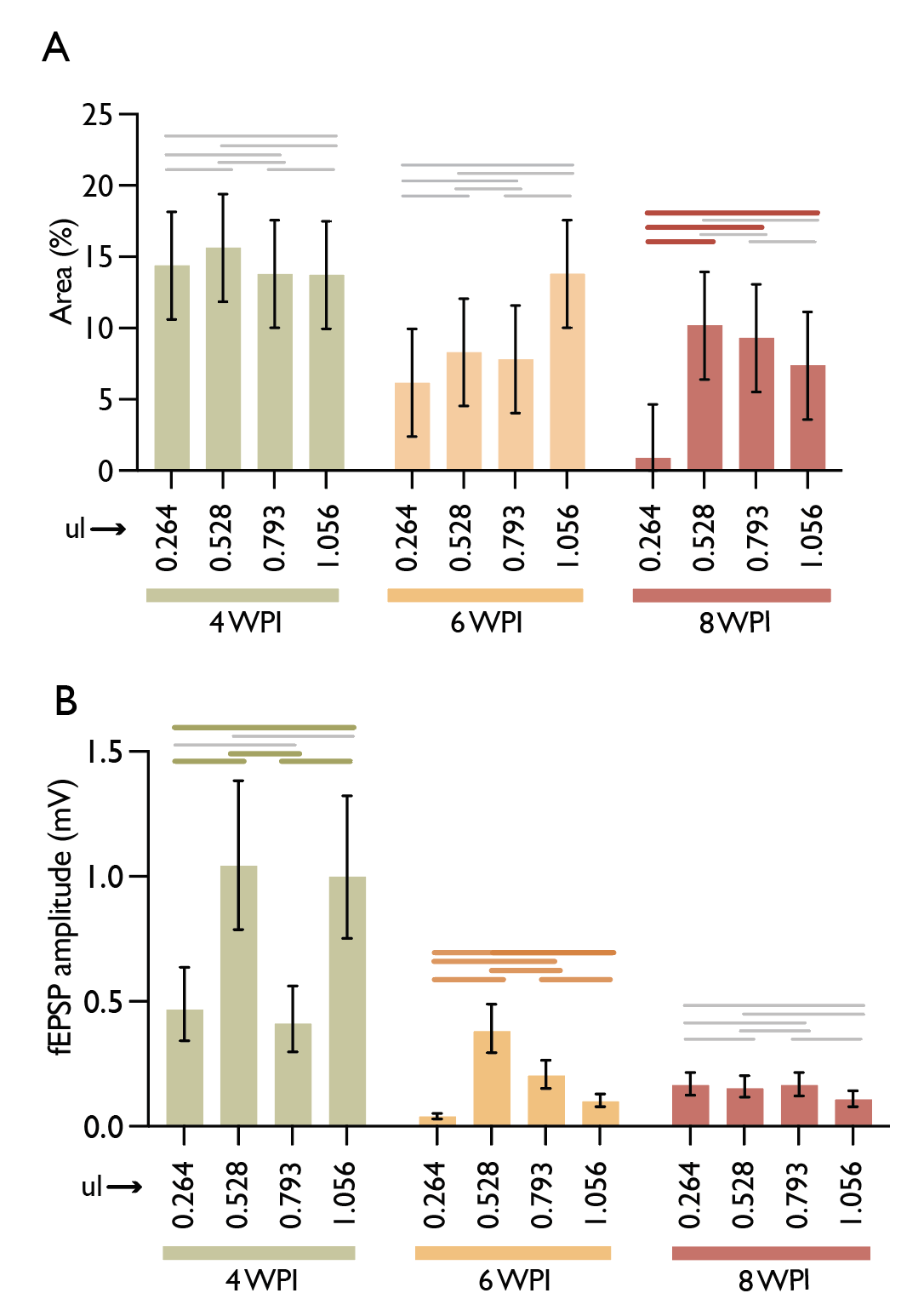


**Supplementary Figure S1**. Multiple comparisons between volumes on each time point shown (same data as in Figure 1 panels E and F, reorganised to show multiple comparisons). Data are represented as means and 95% coverage confidence intervals. Thick coloured lines represent statistically significant differences at a 5% level of significance. Thin grey lines represent no statistical significance detected.


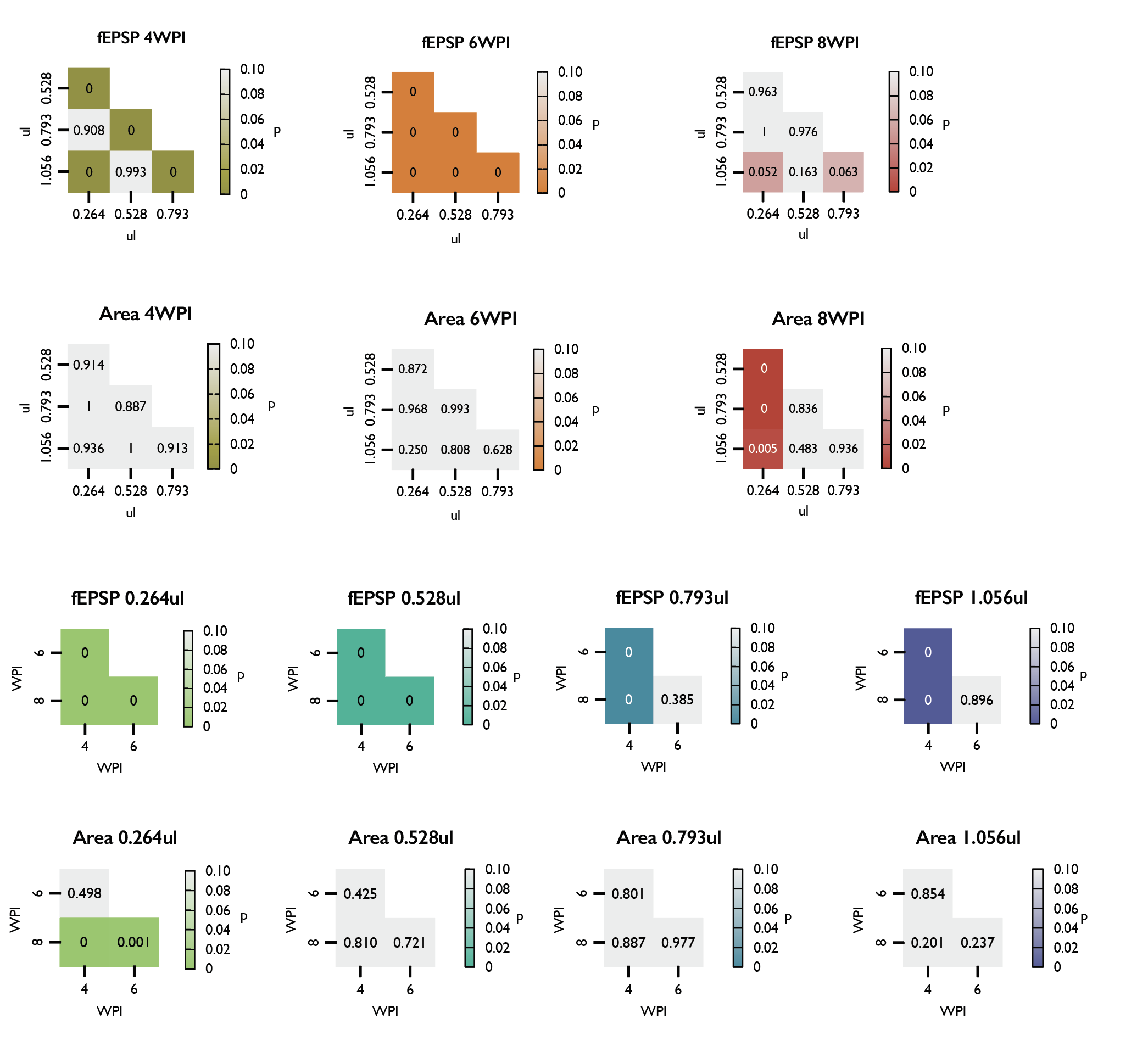


**Supplementary Figure S2**. P-values corresponding to all the comparisons shown in Figure 1 and Supplementary Figure S1. The value 0 is given to p<0.001.

## References

1. Nazari M. Block Artifact Removal [Internet]. Github. 2023.

2. Team RC. R: A language and environment for statistical computing. Vienna, Austria: R Foundation for Statistical Computing; 2022.

3. Bates D, Mächler M, Bolker BM, Walker SC. Fitting linear mixed-effects models using lme4. J Stat Softw. 2015;67(1).

4. Laboriau R. _postHoc: Tools for Post-Hoc Analysis_. [Internet]. R package version 0.1.3; 2020.

5. Benjamini Y, Yekuteli D. The control of the false discovery rate in multiple testing under dependency. Ann Stat. 2001;29(4):1165–88.
